# Outcomes of Hepatic Artery-Based Therapies and Systemic Multiagent Chemotherapy in Unresectable Colorectal Liver Metastases: A Systematic Review and Meta-analysis

**DOI:** 10.1245/s10434-024-15187-y

**Published:** 2024-03-19

**Authors:** Kavin Sugumar, Henry Stitzel, Victoria Wu, David Bajor, Sakti Chakrabarti, Madison Conces, Lauren Henke, Melissa Lumish, Amit Mahipal, Amr Mohamed, Jordan M. Winter, Jeffrey M. Hardacre, John B. Ammori, Jennifer E. Selfridge, Lee M. Ocuin

**Affiliations:** 1 Department of Surgery, Tulane University School of Medicine, New Orleans, LA USA; 2 Case Western Reserve University School of Medicine, Cleveland, OH USA; 3 Division of Hematology/Oncology, Department of Medicine, University Hospitals Seidman Cancer Center, case Western Reserve University, Cleveland, OH USA; 4 Department of Radiation Oncology, University Hospitals Seidman Cancer Center, Cleveland, OH USA; 5 Division of Surgical Oncology, Department of Surgery, University Hospitals Cleveland Medical Center, Cleveland, OH USA

## Abstract

**Background:**

Treatment of unresectable colorectal liver metastases (UCRLM) includes locoregional and systemic therapy. A comprehensive analysis capturing long-term outcomes of these treatment options has not been performed.

**Objective:**

A systematic review and meta-analysis was performed to calculate pooled outcomes of hepatic artery infusion with systemic chemotherapy (HAI-S), transarterial chemoembolization with systemic chemotherapy (TACE-S), transarterial radioembolization with systemic chemotherapy (TARE-S), doublet (FOLFOX, FOLFIRI), and triplet chemotherapy (FOLFOXIRI).

**Methods:**

Outcomes included overall survival (OS), progression-free survival (PFS), rate of conversion to resection (CTR), and response rate (RR).

**Results:**

A total of 32, 7, 9, and 14 publications were included in the HAI-S, TACE-S, and TARE-S chemotherapy arms. The 6/12/24/36-month OS estimates for HAI-S, TACE-S, TARE-S, FOLFOX, FOLFIRI, and FOLFOXIRI were 97%/80%/54%/35%, 100%/83%/40%/14%, 82%/61%/34%/21%, 96%/83%/53%/36%, and 96%/93%/72%/55%. Similarly, the 6/12/24/36-month PFS estimates were 74%/44%/19%/14%, 66%/20%/9%/3%, 57%/23%/10%/3%, 69%/30%/12%/7%, and 88%/55%/18%/11%. The corresponding CTR and RR rates were 31, 20%, unmeasurable (TARE-S), 35, 53; and 49, 45, 45, 50, 80%, respectively. The majority of chemotherapy studies included first-line therapy and liver-only metastases, whereas most HAI-S studies were pretreated. On subgroup analysis in first-line setting with liver-only metastases, the HAI-S arm had comparable outcomes to FOLFOXIRI and outperformed doublet chemotherapy regimens. Although triplet chemotherapy appeared to outperform other arms, high toxicity and inclusion of potentially resectable patients must be considered while interpreting results.

**Conclusions:**

HAI-S and multiagent chemotherapy are effective therapies for UCRLM. To make definitive conclusions, a randomized trial with comparable patient characteristics and line of therapy will be required. The upcoming EA2222 PUMP trial may help to address this question.

**Supplementary Information:**

The online version contains supplementary material available at 10.1245/s10434-024-15187-y.

Colorectal cancer (CRC) is the third most common cancer in males and females and fourth-leading cause of cancer mortality worldwide.^1^ The liver is the most common, and often only, site of metastases, which occur in nearly 50% of the patients.^2^ Resection of colorectal liver metastases confers the best chance for long-term survival, but only 15–20% of patients with colorectal liver metastasis (CRLM) have resectable disease at diagnosis.^3–5^ Importantly, only a fraction of these patients are formally evaluated by a trained hepatobiliary surgical oncologist.^6–8^

Multiagent chemotherapy with or without biological agents, including antiepithelial growth factor receptor therapy and antivascular endothelial growth factor, is the standard first-line option for most patients with unresectable or potentially resectable CRLM.^9^ Standard doublet chemotherapy includes FOLFOX (folinic acid, 5-fluorouracil, oxaliplatin) or FOLFIRI (folinic acid, 5-flourouracil, irinotecan). Patients treated with these regimens have reported conversion rates between 9 and 33%.^10^ Patients treated with the triplet regimen FOLFOXIRI (folinic acid, 5-flourouracil, oxaliplatin, irinotecan) have associated conversion rates of 12–61%, median progression-free survival (PFS) of 7–18 months, and overall survival (OS) of 16.7–27.4 months but experience considerably higher toxicity.^11–13^ Among studies evaluating chemotherapy for unresectable CRLM (UCRLM), there is inconsistency in the reporting and definition of resectability criteria, which makes interpretation of results difficult.^14^

Several hepatic artery-based therapies (HABT) for UCRLM, including hepatic artery infusion (HAI), transarterial chemoembolization (TACE), and transarterial radioembolization (TARE), have been studied in pretreated patients as well as the first-line setting for UCRLM. Hepatic artery infusion involves surgical implantation of a subcutaneous pump with infusion of floxuridine (FUDR), generally combined with systemic chemotherapy (HAI-S), and has shown response and conversion rates as high as 92 and 52%, respectively.^15,16^ TARE utilizes Ytrrium-90 (Y^90^) and delivers high-dose beta-radiation to induce tumor necrosis. The SIRLOX, FOXFIRE, and FOXFIRE-Global trials did not show improved outcomes with addition of Y^90^ to chemotherapy in patients with UCRLM.^17^ TACE involves drug delivery and embolization of agents into tumor-feeding arteries to enable prolonged chemotherapy exposure. Patients with UCRLM treated with TACE have median OS of 9–25 months and PFS of 5–8 months.^18^

At present, there is no consensus on the effectiveness of various HABTs compared to modern multiagent chemotherapy in UCRLM. Herein, we conduct a comprehensive systematic review and meta-analysis to report the pooled outcomes of HABT and modern multiagent chemotherapy in patients with UCRLM. Our primary outcomes were OS and PFS. Secondary outcomes included response rate (RR) and conversion to resection (CTR).

## Methods

### Protocol and Registration

This systematic review and meta-analysis was performed and reported in accordance with Preferred Reporting Items for Systematic Reviews and Meta-analyses statement (PRISMA).^19^ The study protocol was registered in the International Prospective Register of Systematic Reviews (ID: CRD42023410490).

### Search Strategy

A systematic literature search was performed in the Medline database to identify pertinent publications published between January 1, 2003, and April 1, 2023. Four separate literature searches were performed for (1) HAI with systemic chemotherapy, (2) TACE with systemic chemotherapy (TACE-S), (3) TARE with systemic chemotherapy (TARE-S), and (4) systemic multiagent chemotherapy with or without targeted therapy. The complete search strategy is shown in Supplemental Table 1. Studies published in non-English languages were excluded.

**Table 1:** Study characteristics

Study group	Hepatic artery infusion with systemic chemotherapy (HAI-S)	Trans-arterial chemoembolization with systemic chemotherapy (TACE-S)	Trans-arterial radioembolization with systemic chemotherapy (TARE-S)	Multiagent chemotherapy* (CT)
No. studies	32	7	9	14
No. cohorts	38	9	11	21
Type of study				
Retrospective cohort	20 (62.5%)	4 (57.1%)	5 (55.5%)	2 (18.2%)
Clinical trial	12 (37.5%)	3 (42.9%)	4 (44.5%)	12 (81.8%)
Median age (years)	58	62	62	59
Chemotherapy				
FOLFOX, FOLFIRI	–	–	–	17 (76.5%)
FOLFOXIRI	–	–	–	4 (23.5%)
Year of publication	2005–2022	2006–2021	2004–2021	2013–2021
First-line treatment	9 (23.7%)	2 (28.6%)	4 (44.4%)	16 (76.2%)
No extrahepatic disease (EHD)	27 (71.1%)	4 (57.2%)	6 (66.7%)	15 (71.4%)
Outcomes reported				
Overall survival	38 (100%)	9 (100%)	6 (54.5%)	19 (90.5%)
Progression-free survival	18 (47.4%)	4 (44.4%)	6 (54.5%)	15 (71.4%)
Response rate	28 (73.7%)	9 (100%)	6 (54.5%)	16 (76.2%)
Conversion to surgery	20 (52.6%)	3 (33.3%)	1 (9.1%)	18 (85.7%)

### Inclusion and Exclusion Criteria

Studies met the following criteria for inclusion: (1) patients aged 18 years or older who were diagnosed with unresectable colorectal liver metastases; (2) observational cohort studies or clinical trials; (3) patients treated with either HAI therapy with systemic chemotherapy, TACE with systemic chemotherapy, TARE with systemic chemotherapy, or modern multiagent chemotherapy; (4) multiagent chemotherapy regimens included FOLFOX, FOLFIRI, and FOLFOXIRI; (5) details regarding survival, including OS, PFS, conversion to surgical resection, and response rate, were available. Exclusion criteria included: (1) case reports and case series; (2) patients treated with HAI without concurrent systemic chemotherapy; (3) studies using HABT including historical cohorts before 1998; (4) chemotherapy studies including patients with CRM other than liver. HAI without systemic chemotherapy was not included in this meta-analysis, because it has been replaced with HAI-S in most cancer centers because of generally better outcomes in the latter.^14^ To ensure consistency in comparison with HAI-S, TACE and TARE studies that did not use systemic chemotherapy were excluded. We also excluded studies with historical cohorts before 25 years (1998) to reduce the risk of comparing outdated techniques in HAI-S, TACE-S, and TARE-S arms. The year 1998 was chosen deliberately to exclude outdated practices. Three individuals (KS, HS, and VW) independently reviewed titles and abstracts from the above-mentioned databases and selected relevant publications. Studies fulfilling the inclusion criteria and abstracts lacking clear description of study parameters were acquired for a complete-text evaluation. Any disagreement on eligibility for inclusion were reconciled by thorough discussion.

### Data Extraction and Synthesis

Data extracted from studies included sample size, median age, gender, line of therapy, HAI, chemotherapy, chemoembolization, and radioembolization regimens or dose used, line of therapy used, median follow-up period, median OS, PFS, CTR, and RR following treatment completion. The treatment groups included HAI-S, TACE-S, TARE-S, and chemotherapy. The chemotherapy arm was analyzed as two separate groups: FOLFOX/FOLFIRI and FOLFOXIRI due to inherently different toxicity profiles. In the treatment arms, up to one third of studies included patients receiving first-line therapy exclusively, whereas the remaining two thirds of studies included heterogenous cohorts of first-line and pretreated patients. Thus, further stratification of analysis was performed as either first-line or not first-line therapy. For the latter, it was not possible to differentiate second- from third-line therapy in the meta-analysis given the inclusion of patients who failed multiple lines of therapy. The rate of CTCAE (Common Terminology Criteria for Adverse Events)^20^ grade 3–4 toxicities associated with therapy also was recorded.

### Primary Outcomes

The OS and PFS survival estimates at 6, 12, 24, and 36 months were the primary outcomes. Kaplan-Meier (KM) curves for OS and PFS from the individual studies were digitalized by using Webplot digitalizer software.^21^ The survival probabilities at the above timepoints were recorded. OS and PFS were recorded from the start of treatment. The survival estimates of each publication were weighted as a function of the inverse variance of each effect size, and forest plots were constructed. Cochrane *χ*^2^ and *I*^2^ statistics were used to assess homogeneity for each outcome. Studies were considered to have significant heterogenicity when the *χ*^2^ *p* value < 0.1 and *I*^2^ > 50%. The pooled survival probabilities of the treatment groups were calculated either by using the fixed effects model/Manzel-Haenzel method or random effects model/DerSimonian-Laird method based on heterogeneity of the included studies. Random effects method was used when *I*^2^ > 50%.

### Secondary Outcomes

Similarly, the weighted CTR and RR were calculated as a function of the inverse variance of each effect size, and forest plots were constructed.

### Bias and Certainty of Evidence

The ROBINS-I tool was used to ascertain the quality of nonrandomized studies and to determine the risk of bias.^22^ The ROBINS-I tool graded studies into one of the risk of bias categories based on seven areas of potential bias: low risk, some concern, uncertain, and high risk of bias. The GRADE approach was utilized to evaluate the quality of evidence of the meta-analysis. The assessment includes risk of bias, imprecision, inconsistency, indirectness, publication bias, magnitude of effects, dose-response relations, and impact of residual confounding and bias.^23^ Using the above parameters, the GRADE certainty rating is graded as very low, low, moderate, and high. The GRADEpro Guideline Development Tool (McMaster University, 2020, developed by Evidence Prime, Inc.) was used to calculate and tabulate the GRADE certainty rating.

All statistical analyses were performed with StataSE Version 16 software (StataCorp. 2021. Stata Statistical Software: Release 16. College Station, TX: StataCorp LLC).

## Results

**Fig. 1:**
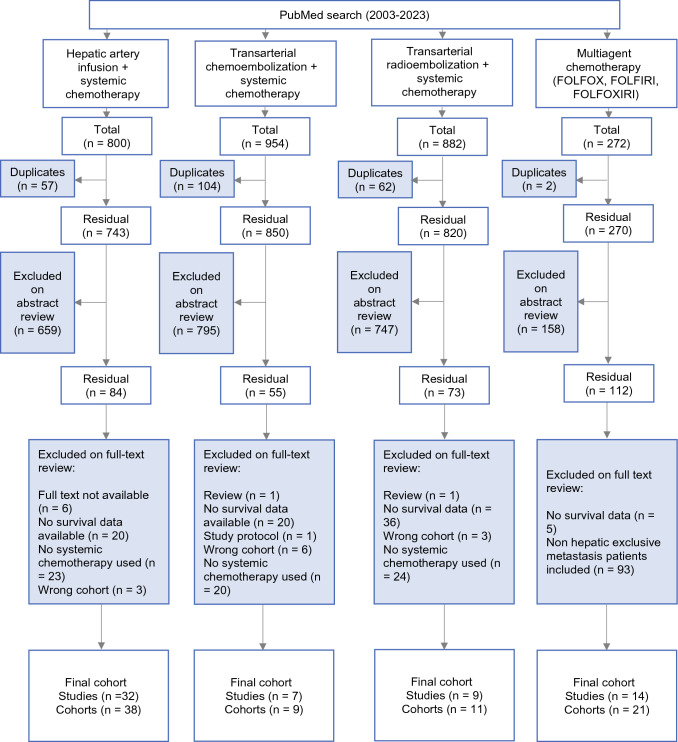
PRISMA study flow diagram

Our literature search yielded 800, 954, 882, and 272 publications in the HAI-S, TACE, TARE, and chemotherapy arms, respectively. Figure 1 summarizes reasons for exclusion. The main reasons for exclusion in the HAI-S arm were studies published before 2010 that did not utilize concurrent systemic chemotherapy. In the chemotherapy arm, 93 studies were excluded due to inclusion of patients with extrahepatic metastases or metastatic primary sites other than colorectal cancer. On systematic review, 32, 9, 11, and 14 studies met inclusion criteria in the HAI-S, TACE-S, TARE-S, and chemotherapy arms and were included in this meta-analysis.

### Study Characteristics

Study characteristics are shown in Table 1. Nearly one-third of the included studies had more than one cohort that were used in the meta-analysis. The chemotherapy arm comprised the highest proportion of clinical trials (81.8%) compared with the HAI, TACE-S, and TARE-S arms (37.5, 42.9, 44.5%). In the chemotherapy arm, nearly two thirds of studies used FOLFOX or FOLFIRI. Most of the chemotherapy arm studies included first-line therapies (76.2%), whereas HABT was utilized more often as second- or third-line therapy. Studies included patients with no extrahepatic disease (EHD) in 71.1, 57.2, 66.7, and 71.4% of cohorts in the four arms. CTR was less commonly reported; up to half of the HAI-S, TACE-S, and TARE-S arms reported CTR compared with 85.7% in the chemotherapy arm.

### Overall Survival

**Fig. 2:**
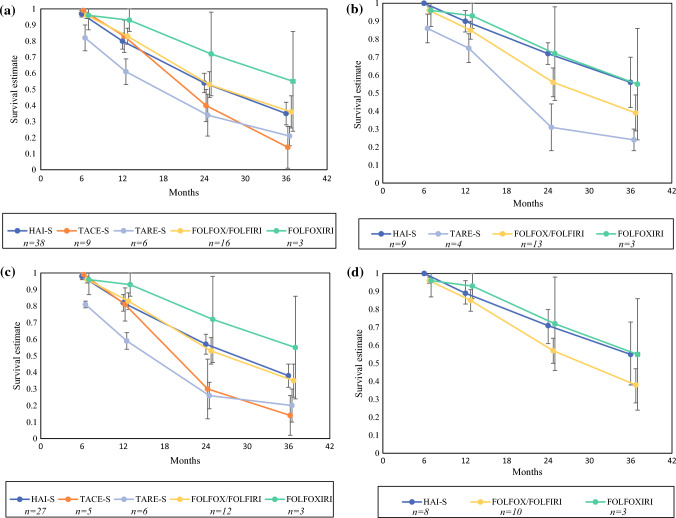
Pooled overall survival estimates at 6, 12, 24, and 36 months in: **a** all cohorts; **b** first-line therapy; **c** no extrahepatic disease; **d** first-line therapy in patients without extrahepatic disease. *HAI-S* hepatic artery infusion with systemic chemotherapy; *TACE-S* transarterial chemoembolization with systemic chemotherapy; *TARE-S* transarterial radioembolization with systemic chemotherapy; *FOLFOX* folinic acid, 5-flourouracil, oxaliplatin; *FOLFIRI* folinic acid, 5-fluorouracil, irinotecan; *FOLFOXIRI* folinic acid, 5-fluorouracil, oxaliplatin, irinotecan; *n* number of cohorts

Using Forest plot analysis, the pooled 6-month, 12-month, 24-month, and 36-month OS survival estimates were calculated for the treatment arms (Supplemental Table 2; Fig. 2). Subgroup analysis was performed for studies, including first-line therapy only, no EHD, and a combination of both parameters. Analysis was not performed if pooled analysis included fewer than three cohorts. For the HAI-S arm, the OS estimates at 6, 12, 24, and 36 months were 97, 80, 54, and 35%. Among studies using first line-therapy only and patients with no EHD, the survival probabilities were better (100, 89, 71, 55%). For TACE-S, the survival estimates at 6, 12, 24, and 36 months were 100, 83, 40, and 14%. For TARE-S, the survival estimates were 82, 61, 34, and 21%. For the chemotherapy arm, patients who received FOLFOX or FOLFIRI arm had survival probabilities of 96, 83, 53, and 36%. Patients treated with FOLFOXIRI had survival probabilities of 96, 93, 72, and 55%. Among patients with first-line therapy and no EHD, the survival probabilities of the chemotherapy groups were similar, as majority of these studies included first-line therapy in CRLM without EHD (96, 85, 57, 38% [FOLFOX/FOLFIRI], and 96, 93, 72, 55% [FOLFOXIRI]).

### Progression-free Survival

**Fig. 3:**
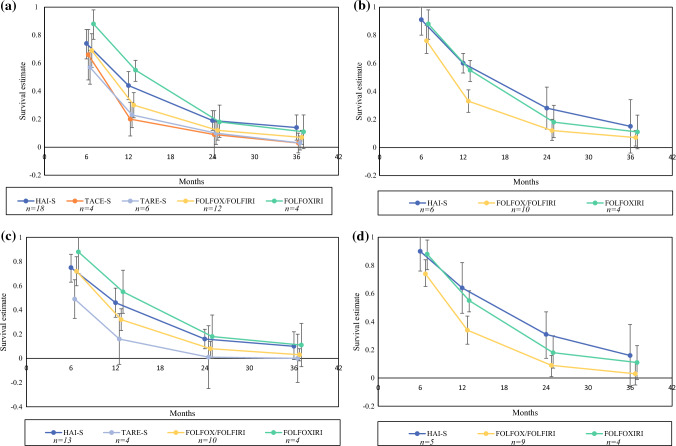
Pooled progression-free survival estimates at 6, 12, 24, and 36 months in: **a** all cohorts; **b** first-line therapy; **c** no extrahepatic disease; **d** first-line therapy in patients without extrahepatic disease. *HAI-S* hepatic artery infusion with systemic chemotherapy; *TACE-S* transarterial chemoembolization with systemic chemotherapy; *TARE-S* transarterial radioembolization with systemic chemotherapy; *FOLFOX* folinic acid, 5-flourouracil, oxaliplatin; *FOLFIRI* folinic acid, 5-fluorouracil, irinotecan; *FOLFOXIRI* folinic acid, 5-fluorouracil, oxaliplatin, irinotecan; *n* number of cohorts

Using forest plot analysis, the pooled 6-month, 12-month, 24-month, and 36-month PFS survival estimates were calculated for the treatment arms (Supplemental Table 3; Fig. 3). Subgroup analysis was performed for studies including first-line therapy only, patients with no EHD, and a combination of both parameters. Analysis was not performed if pooled analysis included less than three cohorts. For the HAI-S arm, the PFS at 6, 12, 24, and 36 months were 74, 44, 19, and 14%. Among studies using first line-therapy only and patients with no EHD, the survival estimates were better (90, 64, 31, 16%). For TACE-S, the PFS at 6, 12, 24, and 36 months was 66, 20, 9, and 3%. For TARE-S, the PFS was 57, 23, 10, and 3%. The FOLFOX/FOLFIRI arm had survival probabilities of 69, 30, 12, and 7%. Patients treated with FOLFOXIRI had survival estimates of 88, 55, 18, and 11%. Among patients with first-line therapy and no EHD, the survival estimates for FOLFOX/FOLFIRI were 74, 34, 9, and 3% and for FOLFOXIRI were 88, 55, 18%, and 11%.

### Conversion to Resection Rate

**Fig. 4:**
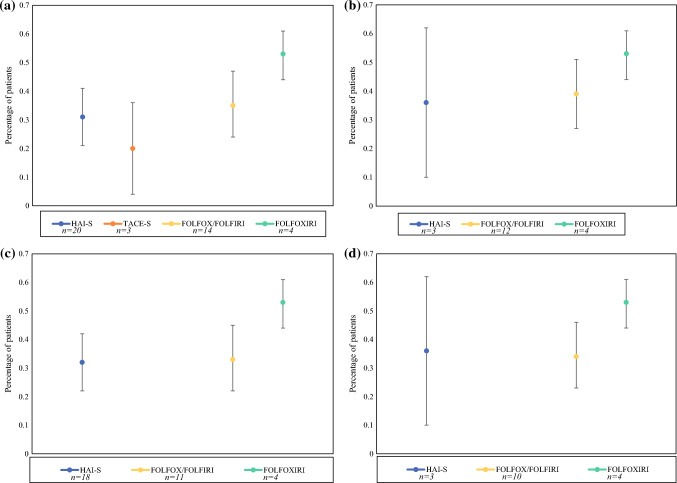
Pooled rates of conversion to resection in **a** all cohorts; **b** first-line therapy; **c** no extrahepatic disease; **d** first-line therapy in patients without extrahepatic disease. *HAI-S* hepatic artery infusion with systemic chemotherapy; *TACE-S* transarterial chemoembolization with systemic chemotherapy; *TARE-S* transarterial radioembolization with systemic chemotherapy; *FOLFOX* folinic acid, 5-flourouracil, oxaliplatin; *FOLFIRI* folinic acid, 5-fluorouracil, irinotecan; *FOLFOXIRI* folinic acid, 5-fluorouracil, oxaliplatin, irinotecan; *n* number of cohorts

The pooled CTR analysis is shown in Supplemental Table 4 and Fig. 4. Subgroup analysis was performed for studies including first-line therapy only, no EHD, and a combination of both. Analysis was not performed if pooled analysis included less than three cohorts. The pooled conversion rates for HAI-S, TACE-S, FOLFOX/FOLFIRI, and FOLFOXIRI arms were 31, 20, 35, and 53% respectively. Meta-analysis for TARE-S was not possible, because only one study reported CTR. Among studies using first-line therapy and no EHD, the pooled CTR for HAI-S, FOLFOX/FOLFIRI, and FOLFOXIRI arms were 36, 34, and 53% respectively.

### Response Rate

**Fig. 5:**
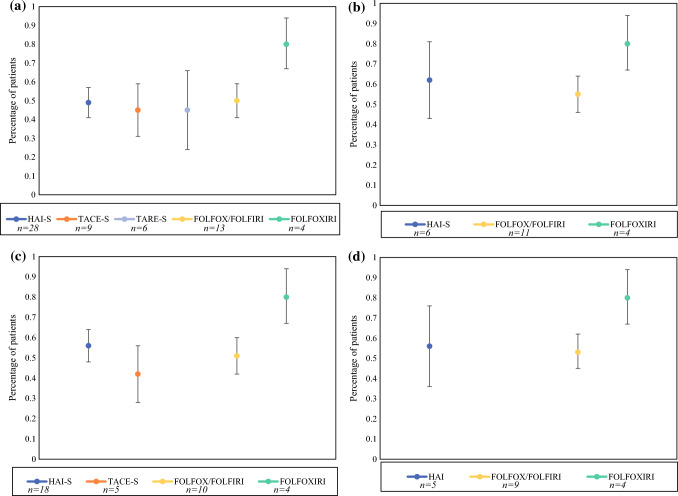
Pooled response rates in **a** all cohorts; **b** first-line therapy; **c** no extrahepatic disease; **d** first-line therapy in patients without extrahepatic disease. *HAI-S* hepatic artery infusion with systemic chemotherapy; *TACE-S* transarterial chemoembolization with systemic chemotherapy; *TARE-S* transarterial radioembolization with systemic chemotherapy; *FOLFOX* folinic acid, 5-flourouracil, oxaliplatin; *FOLFIRI* folinic acid, 5-fluorouracil, irinotecan; *FOLFOXIRI* folinic acid, 5-fluorouracil, oxaliplatin, irinotecan; *n* number of cohorts

The pooled RR analysis is shown in Supplemental Table 4 and Fig. 5. Subgroup analysis was performed for studies, including first-line therapy only, no EHD, and a combination of both. Analysis was not performed if the pooled analysis included less than three cohorts. The pooled RR for HAI-S, TACE-S, TARE-S, FOLFOX/FOLFIRI, and FOLFOXIRI arms were 49, 45, 45, 50, and 80%. Among studies using first-line therapy and no EHD, the pooled RR for HAI-S, FOLFOX/FOLFIRI, and FOLFOXIRI groups were 56%, 53%, and 80%.

### Risk of Bias Analysis

**Fig. 6:**
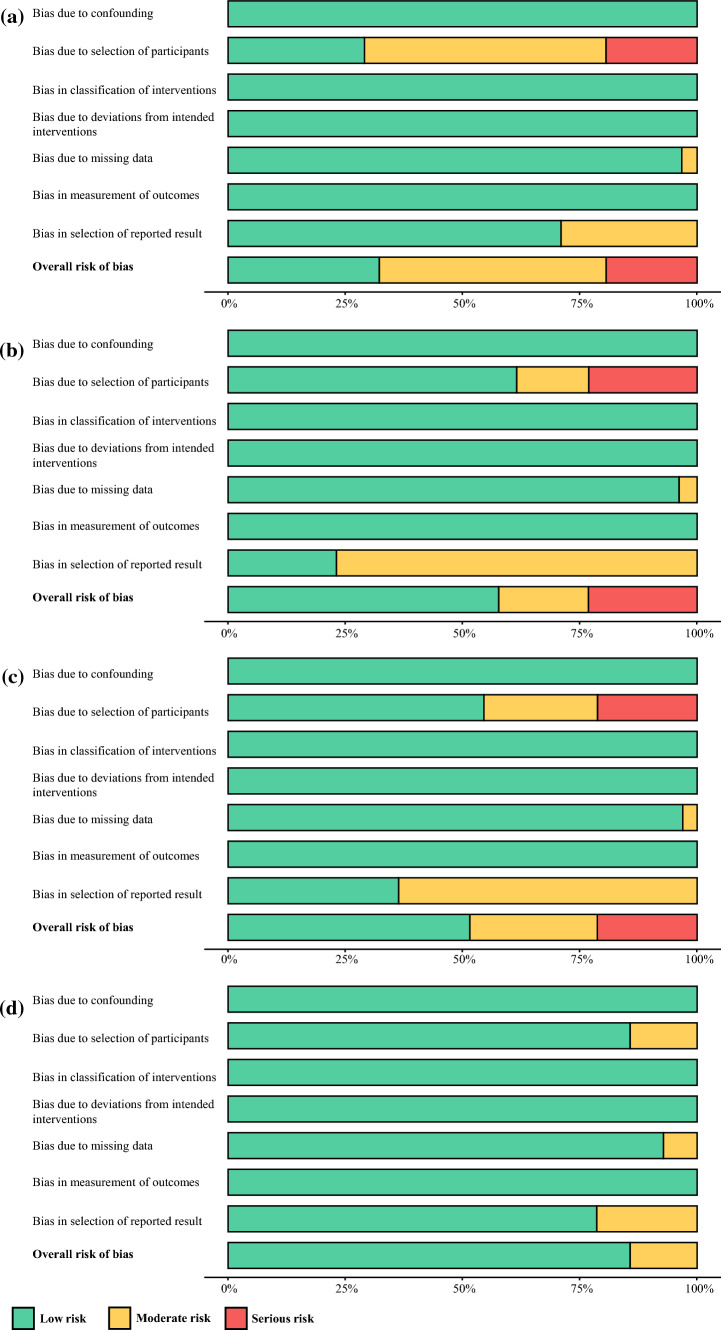
Risk of bias summary graph using ROBINS-I tool. **a** *HAI-S* hepatic artery infusion; **b** *TACE-S* transarterial chemoembolization with systemic chemotherapy; **c** *TARE-S* transarterial radioembolization with systemic chemotherapy; **d** *CT* multiagent chemotherapy

The risk of bias analysis calculated using the ROBINS-I tool is described in Fig. 6 and Supplemental Tables 5–8. Overall, the included studies had low risk of bias. The most significant source of bias was selection bias; nearly one fourth of HAI-S, TACE-S, and TARE-S cohorts had sample sizes of less than 25 patients. The other source of bias was bias in reporting; nearly half of HAI-S, TACE-S, and TARE-S studies did not report CTR or RR.

### GRADE Certainty of Evidence

The GRADE score of this meta-analysis is shown in Supplemental Table 9. Provided the retrospective study design and low sample size in nearly two thirds of the included HAI-S, TACE-S, and TARE-S studies and associated high risk of selection bias, the certainty of evidence was low. Conversely, majority of chemotherapy studies were prospective, randomized clinical trials with overall low risk of bias. The certainty of evidence of the chemotherapy arm was moderate.

## Discussion

Limited evidence exists comparing regional hepatic artery therapies with systemic multiagent chemotherapy in UCRLM. Our meta-analysis provides an overview of long-term outcomes of various treatment options that have been previously investigated. Although it is not possible to compare outcomes between the treatment arms directly given the nature of the data and included studies, our results provide a concise summary of all previously published data on HABTs compared with modern chemotherapy in the past 20 years. Descriptive statistical analysis presented here provides a snapshot of the key characteristics and patterns of the available data in visual format, which may not be apparent from raw data from several studies. It may act as an aid in decision making among clinicians when evaluating and counseling individual patients on systemic and/or liver-directed therapeutic options by providing a comprehensive descriptive analysis of each treatment modality.

On subgroup analysis, after excluding cohorts with pretreated patients and/or EHD, the HAI-S, TACE-S, and TARE-S arms were associated with better OS especially at 24 and 36 months. However, the doublet (FOLFOX, FOLFIRI) and triplet arms (FOLFOXIRI) was associated with similar OS estimates on exclusion of the pretreated patients and/or EHD, because the majority of the studies were first-line therapies and only included patients with no EHD. As first-line therapy in patients with no EHD, HAI-S showed promising outcomes. More than 50% of patients survived 3 years and up to 50% did not show disease progression until 1–2 years after initiating therapy. The pooled OS outcomes of HAI-S could be associated with patient selection. Most of the HAI-S, TACE-S, and TARE-S studies were single-institution, retrospective, single-arm studies. The pooled OS outcomes of FOLFOXIRI also were promising. However, given the higher toxicity profile and marginal improvement in survival and CTR compared with doublet regimens, broader utilization in patients with UCRLM needs to be further evaluated and currently remains limited to the highest-performing patients.^24^

HAI without systemic chemotherapy was first evaluated in the 1970s. Many studies have compared HAI alone versus single-agent chemotherapy. The CALBG 9481 trial demonstrated efficacy of FUDR-based HAI compared with systemic 5-FU in patients with URCLM, with improved median OS (24 vs. 20 months, *p* = 0.0034) and RR (47 vs. 24%, *p* = 0.12).^25^ A meta-analysis of randomized clinical trials published in 2007 showed that HAI alone had better RR compared with systemic single-agent chemotherapy (42.9 vs. 18.4%). However, this did not translate to improved OS (hazard ratio [HR] 0.90; 95% confidence interval [CI] 0.76–1.07).^26^ HAI therapy in patients with UCRLM has evolved to include combined systemic chemotherapy, with improved outcomes compared with HAI alone, and is therefore the preferred regimen in modern-day clinical practice.^14,27^ Several single-arm trials have evaluated HAI-S in the setting of UCRLM, but it has not been compared with multiagent systemic chemotherapy in a prospective, randomized clinical trial to date. Dhir et al. performed a retrospective case-control analysis comparing HAI-S to systemic FOLFOX or FOLFIRI ± bevacizumab in pretreated patients with comparable tumor burden (median 13.5 vs. 15 liver metastases) and reported that HAI-S was associated with improved median OS (32.8 vs. 15.3 months, *p* < 0.0001; HR 0.4; 95% CI 0.21–0.72).^28^ Our study shows that HAI-S has favorable outcomes, with pooled RR and CTR of 56 and 36% as first-line therapy in liver-only disease patients. The OS and PFS was comparable between HAI-S and FOLFOX/FOLFIRI while including all cohorts, keeping in mind that the majority of the chemotherapy studies were in the first-line setting and the majority of HAI-S studies included pretreated patients. In the first-line setting, HAI appeared to have superior survival outcomes at 24 and 36 months compared with doublet chemotherapy but not with triplet chemotherapy. The upcoming ECOG-ACRIN trial (EA2222/The PUMP Trial; NCT05863195) will compare efficacy of HAI-S to chemotherapy in patients with UCRLM.

Other hepatic, artery-based treatments rarely have been compared with systemic chemotherapy in UCRLM. Fiorentini et al. conducted a randomized phase III trial comparing irinotecan-loaded drug-eluting beads (DEBIRI-TACE) and systemic FOLFIRI in pretreated patients with liver-only disease. DEBIRI-TACE had better OS (22 vs. 15 months, *p* = 0.031), PFS (7 vs. 4 months, *p* = 0.006), and better patient-reported quality of life.^29^ Similarly, Liu et al. performed a single-institution phase III trial and concluded that TACE with chemotherapy had better PFS compared with FOLFOX or FOLFIRI (6.7 vs. 3.8 months, *p* = 0.009) but similar OS (18.4 vs. 14 months, *p* = 0.669).^30^ The FOXFIRE, SIRLOX, and FOXFIRE-Global trials compared TARE with FOLFOX and FOLFOX alone as first-line therapy in liver-only and liver-predominant UCRLM. A pooled multi-institutional analysis of the three trials showed that addition of TARE to FOLFOX did not improve OS or PFS.^17^ In concordance with these data, our meta-analysis of TARE-S studies showed poor survival, with median OS of just over a year and median PFS of approximately 6–9 months.

Previous reports have compared hepatic artery-based treatments in UCRLM. Cercek et al. retrospectively compared floxuridine-based HAI and TARE in pretreated UCRLM and did not find an associated difference in OS. However, on subgroup analysis of patients without EHD, HAI had higher median OS (22 vs. 9 months, *p* = 0.004). This analysis was limited by comparing non case-matched patients treated at two separate institutions, leading to significant institutional bias.^31^ Similarly, Dhir et al. showed associated improvement in median OS in patients treated with HAI compared to TARE (31 vs. 16 months).^32^ Mokkarala et al. and Hong et al. retrospectively compared TACE to TARE, reporting comparable survival.^33,34^ In 2015, a meta-analysis of 90 studies compared HAI, TACE, and TARE in unresectable CRLM. The pooled median OS was similar between groups (21.4, 15.4, and 29.4 months respectively).^35^ However, the HAI studies used in the analysis included cohorts who received HAI alone without systemic therapy, which is not the current standard approach. Additionally, the study included historical cohorts from the early HAI experience, which may have contributed to skewed results. Finally, median OS as a pooled measure is a poor metric for comparison of outcomes. Hazard ratio is the ideal statistical comparison for time-to-event analysis in meta-analyses. Compared with median OS, which takes into consideration a single time point on the survival curve, hazard ratios incorporate changes over time between treatment groups.^36^ Because of the noncomparative nature of included studies, it is impossible to estimate hazard ratios of trial-level data. Hence to characterize changes over time, we compared survival probabilities at various timepoints.

Apart from long-term outcomes, it is important to study the associated morbidity of HABT. We report the rate of grade 3–4 adverse effects following HAI-S was variable, ranging from 5 to 69%. The termination rate for HAI-S ranged from 5 to 28%. However, there was significant heterogeneity in reporting of toxicity and morbidity. This made it impossible to perform a worthwhile meta-analysis with meaningful conclusions. Although the addition of chemotherapy appears to improve survival in UCRLM, the added morbidity must be considered during treatment planning. Further research is needed to better characterize the cost-effectiveness and quality of life among patients receiving HAI-S. Similarly, for the TACE arm, the rate of grade 3–4 adverse effects ranged between 2 and 25%. A meta-analysis showed that the pooled rate of grade 3–4 toxicities associated with HAI-S, TACE, and TARE are 55, 17, and 26%, respectively. For HAI alone, the grade 3–4 toxicity rate was lower (40%). The termination rate ranged between 6.5 and 8% compared with 20% in HAI alone and HAI-S subgroups respectively.^35^

The most common multiagent chemotherapy regimens for UCRLM include FOLFOX and FOLFIRI. Addition of targeted therapy to chemotherapy has shown to improve outcomes in such patients.^37–39^ Our meta-analysis shows promising outcomes for FOLFOX/FOLFIRI, with pooled CTR and RR of 35 and 50%, respectively. The higher CTR could be the result of inconsistency in the definition of unresectability of CRLM and inclusion of potentially resectable disease.

FOLFOXIRI also has been investigated in patients with UCRLM. Falcone et al. performed a phase II trial comparing FOLFOXIRI and FOLFIRI in metastatic colorectal cancer. FOLFOXIRI improved RR, OS, and PFS. FOLFOXIRI group had a worse toxicity profile with patients more commonly experiencing grade 2–3 neurotoxicity (19 vs. 0%) and grade 3–4 neutropenia (50 vs. 28%).^11^ Similarly, Khali et al. compared toxicities between FOLFOXIRI and doublet therapy in a phase II trial among patients with metastatic colorectal cancer. FOLFOXIRI had higher grade 3–4 neutropenia (31.3 vs. 6.3%) and febrile neutropenia (12.5 vs. 0%).^40^ A meta-analysis of two trials compared safety and efficacy of first line FOLFOXIRI and FOLFIRI in metastatic colorectal cancer patients. FOLFOXIRI was associated with higher frequency of neutropenia (odds ratio [OR] = 1.85), nausea (OR = 2.68), diarrhea (OR = 2.54), and neurotoxicity (OR = 14.66). FOLFOXIRI had better RR and R0 resection rate.^41^ Another meta-analysis of eight randomized clinical trials showed that FOLFOXIRI had worse grade 3–4 toxicities, including neurological (OR = 8.63), anemia (OR = 2.51), neutropenia (OR = 1.81), and mucositis (OR = 1.76), compared with FOLFOX or FOLFIRI. Toxicity was not uniformly reported across included studies.^42^ Our meta-analysis only included four studies evaluating FOLFOXIRI, showing 3-year OS and PFS survival probabilities of 55% and 11%, respectively, CTR of 53%, and RR of 80%. These results need to be interpreted with caution. Similar to FOLFOX/FOLFIRI studies, half of the included studies evaluating FOLFOXIRI included potentially resectable disease.^43,44^ While the improved outcomes are promising, the added toxicity may limit use to healthier patient populations with better baseline performance status.

Results of this meta-analysis needs to be interpreted considering certain notable limitations. Most of the data from the HABT are single-institutional, retrospective cohort studies with small sample sizes, which can result in significant selection bias and which may affect the overall accuracy of findings and less representative of the general population.^45^ For example, most of HAI-S studies were retrospective, with possibility of selection bias for patients with higher functional status or other pretreatment factors that cannot be determined from this review. To reduce selection bias, an ideal comparison would be a prospective study with comparable patient characteristics, randomization, and use of masking.^45^ Five HABT studies included partially overlapping patient populations, which may reduce the overall precision of results. There was significant heterogeneity among the included studies, including significantly different disease burden, inclusion of patients with varying lines of therapy, presence of extrahepatic disease, different chemotherapy, HAI, and TACE therapy regimens. It was not possible to pool the toxicity profiles of the HABT as well as systemic chemotherapy given inconsistency in reporting. We tried to control for lines of chemotherapy by performing subgroup analysis for those studies investigating first-line therapy. However, we were not able to construct subgroups based on a specific number of lines of therapy before treatment (e.g., second-line, third-line, etc.). We also performed subgroup analyses in patients with liver-only metastatic disease. Most of the studies in the chemotherapy arm added targeted therapy, which may have contributed to better outcomes compared with the HABT studies, which rarely employed them. As previously discussed, the chemotherapy arm included several studies that had patients with potentially or technically resectable disease, which could have altered the results. Because of the above-mentioned limitations, the grade certainty of evidence was low in the HABT compared with a moderate level of evidence in the system of chemotherapy regimens.

## Conclusions

The treatment options for patients with unresectable colorectal liver metastases continues to evolve. Our meta-analysis provides a comprehensive summary of outcomes of various treatment options for these patients. Among patients without extrahepatic disease receiving first-line HAI-S, nearly 50% of patients survive up to 3 years after initiation of therapy, and 50% of patients do not progress until 2 years from therapy initiation. FOLFOXIRI appears to offer promising OS and PFS. TACE-S and TARE-S are associated with poor survival; more than two thirds of patients died by the end of 2 years. Randomized clinical trials in patients with comparable disease burden and lines of therapy are required to compare the long-term outcomes of HAI-S to systemic, multiagent chemotherapy.

### Supplementary Information

Below is the link to the electronic supplementary material.

Supplementary file1 (DOCX 48 kb)

## Data Availability

Data used in this meta-analysis will be available on reasonable request to authors.
