# Supplementary material for: Outcomes of Hepatic Artery-Based Therapies and Systemic Multiagent Chemotherapy in Unresectable Colorectal Liver Metastases: A Systematic Review and Meta-analysis
Source: Ann Surg Oncol. 2024 Mar 19;31(7):4413–26. doi: 10.1245/s10434-024-15187-y (PMC11164761; doi:10.1245/s10434-024-15187-y)
Supplement: Supplementary file 1 — Supplementary file1 (DOCX 48 kb) [file 10434_2024_15187_MOESM1_ESM.docx]

Supplemental Table 1 (online only). Search strategy of treatment arms

| Treatment arms | Medline search strategy |
| --- | --- |
| **Hepatic artery infusion** | (("colorectal neoplasm*"[MeSH Terms:noexp] OR "liver neoplasm*"[MeSH Terms:noexp] OR "colorectal cancer*"[Title/Abstract] OR "liver metastas*"[Title/Abstract] OR "rectum"[Title/Abstract] OR "colon"[Title/Abstract]) AND ("Hepatic artery infusion"[Title/Abstract] OR "Hepatic artery"[MeSH Terms] OR "Infusion Pumps"[MeSH Terms] OR "infusions, intra arterial"[MeSH Terms] OR "infusion pumps, implantable"[MeSH Terms:noexp]) AND ("Survival Rate"[MeSH Terms] OR "overall survival"[Title/Abstract] OR "progression free survival"[Title/Abstract] OR "survival"[Title/Abstract])) AND ((fft[Filter]) AND (english[Filter]) AND (2003:2023[pdat])) NOT Case Reports |
| **Transarterial chemoembolization** | (((("Colorectal Neoplasms"[Mesh] AND "Liver Neoplasms"[Mesh]) AND ("Chemoembolization, Therapeutic"[Mesh] OR "Embolization, Therapeutic"[Mesh:noexp] OR "Hepatic Artery"[Mesh] OR ((((Colorectal[All Fields] OR ("colon"[MeSH Terms] OR "colon"[All Fields]) OR ("rectum"[MeSH Terms] OR "rectum"[All Fields])) AND (("liver"[MeSH Terms] OR "liver"[All Fields]) OR hepatic[All Fields])) AND ((hepatic[All Fields] AND ("arteries"[MeSH Terms] OR "arteries"[All Fields] OR "arterial"[All Fields]) AND chemoembolization[All Fields] OR chemoembolisation[All Fields])) AND (("neoplasm metastasis"[MeSH Terms] OR ("neoplasm"[All Fields] AND "metastasis"[All Fields]) OR "neoplasm metastasis"[All Fields] OR "metastasis"[All Fields]) OR ("secondary"[Subheading] OR "secondary"[All Fields] OR "metastatic"[All Fields]) OR ("neoplasm metastasis"[MeSH Terms] OR ("neoplasm"[All Fields] AND "metastasis"[All Fields]) OR "neoplasm metastasis"[All Fields] OR "metastases"[All Fields]))) AND ("neoplasms"[MeSH Terms] OR "neoplasms"[All Fields] OR "cancer"[All Fields]))) NOT Case reports |
| **Transarterial radioembolization** | (((("Colorectal Neoplasms"[Mesh] AND "Liver Neoplasms"[Mesh]) AND ("Chemoembolization, Therapeutic"[Mesh] OR "Embolization, Therapeutic"[Mesh:noexp] OR "Hepatic Artery"[Mesh] OR "Yttrium Radioisotopes"[Mesh] OR ((((Colorectal[All Fields] OR ("colon"[MeSH Terms] OR "colon"[All Fields]) OR ("rectum"[MeSH Terms] OR "rectum"[All Fields])) AND (("liver"[MeSH Terms] OR "liver"[All Fields]) OR hepatic[All Fields])) AND ((hepatic[All Fields] AND ("arteries"[MeSH Terms] OR "arteries"[All Fields] OR "arterial"[All Fields]) AND radioembolization[All Fields] OR radioembolisation[All Fields])) AND (("neoplasm metastasis"[MeSH Terms] OR ("neoplasm"[All Fields] AND "metastasis"[All Fields]) OR "neoplasm metastasis"[All Fields] OR "metastasis"[All Fields]) OR ("secondary"[Subheading] OR "secondary"[All Fields] OR "metastatic"[All Fields]) OR ("neoplasm metastasis"[MeSH Terms] OR ("neoplasm"[All Fields] AND "metastasis"[All Fields]) OR "neoplasm metastasis"[All Fields] OR "metastases"[All Fields]))) AND ("neoplasms"[MeSH Terms] OR "neoplasms"[All Fields] OR "cancer"[All Fields]))) NOT Case reports |
| **Multiagent chemotherapy** | (("colorectal neoplasms"[MeSH Terms] OR "cholangiocarcinoma"[MeSH Terms] OR "colorectal liver metastases"[Title/Abstract] OR (("liver neoplasms"[MeSH Terms] OR ("liver"[All Fields] AND "neoplasms"[All Fields]) OR "liver neoplasms"[All Fields]) AND "secondary*"[MeSH Terms])) AND "overall survival"[Title/Abstract] AND ("folfox protocol"[Supplementary Concept] OR "IFL protocol"[Supplementary Concept] OR "folfoxiri"[Supplementary Concept]) AND "antibodies, monoclonal, humanized"[MeSH Terms]) AND ((2003:2023[pdat]) AND (english[Filter])) |

Supplemental Table 2 (online only). Pooled estimates for overall survival

| **Overall Survival** | **Number of cohorts** | **6-month** | **12-month** | **24-month** | **36-month** |
| --- | --- | --- | --- | --- | --- |
| **All cohorts** |  |  |  |  |  |
| HAI-S | 38 | 0.97 (0.96-0.98) | 0.80 (0.75-0.84) | 0.54 (0.48-0.61) | 0.35 (0.28-0.42) |
| TACE-S | 9 | 1 (0.99-1) | 0.83 (0.73-0.93) | 0.40 (0.14-0.67) | 0.14 (0.01-0.26) |
| TARE-S | 6 | 0.82 (0.74-0.89) | 0.61 (0.43-0.79) | 0.34 (0.21-0.47) | 0.21 (0.15-0.27) |
| FOLFOX, FOLFIRI | 16 | 0.96 (0.94-0.97) | 0.83 (0.78-0.88) | 0.53 (0.45-0.61) | 0.36 (0.26-0.45) |
| FOLFOXIRI | 3 | 0.96 (0.87-1.4) | 0.93 (0.80-1.3) | 0.72 (0.46-0.99) | 0.55 (0.24-0.86) |
| **First-line only** |  |  |  |  |  |
| HAI-S | 9 | 1 (0.99-1) | 0.90 (0.84-0.96) | 0.72 (0.64-0.80) | 0.56 (0.40-0.71) |
| TACE-S | 2 | * | * | * | * |
| TARE-S | 4 | 0.86 (0.83-0.89) | 0.75 (0.71-0.79) | 0.31 (0.26-0.37) | 0.24 (0.17-0.31) |
| FOLFOX, FOLFIRI | 13 | 0.96 (0.94-0.98) | 0.85 (0.80-0.91) | 0.56 (0.48-0.64) | 0.39 (0.29-0.49) |
| FOLFOXIRI | 3 | 0.96 (0.87-1.4) | 0.93 (0.80-1.3) | 0.72 (0.46-0.99) | 0.55 (0.24-0.86) |
| **No EHD** |  |  |  |  |  |
| HAI-S | 27 | 0.98 (0.97-0.99) | 0.82 (0.78-0.87) | 0.57 (0.50-0.63) | 0.38 (0.30-0.45) |
| TACE-S | 5 | 0.99 (0.98-1) | 0.81 (0.71-0.91) | 0.30 (0.12-0.48) | 0.14 (0.02-0.26) |
| TARE-S | 6 | 0.81 (0.73-0.90) | 0.59 (0.42-0.77) | 0.26 (0.15-0.37) | 0.20 (0.08-0.30) |
| FOLFOX, FOLFIRI | 12 | 0.96 (0.95-0.98) | 0.83 (0.77-0.89) | 0.53 (0.45-0.61) | 0.35 (0.26-0.44) |
| FOLFOXIRI | 3 | 0.96 (0.87-1.4) | 0.93 (0.80-1.3) | 0.72 (0.46-0.99) | 0.55 (0.24-0.86) |
| **No EHD, first-line only** |  |  |  |  |  |
| HAI-S | 8 | 1 (0.99-1) | 0.89 (0.83-0.96) | 0.71 (0.61-0.80) | 0.55 (0.38-0.73) |
| TACE-S | 1 | * | * | * | * |
| TARE-S | 2 | * | * | * | * |
| FOLFOX, FOLFIRI | 10 | 0.96 (0.95-0.98) | 0.85 (0.79-0.91) | 0.57 (0.5-0.64) | 0.38 (0.30-0.47) |
| FOLFOXIRI | 3 | 0.96 (0.87-1.4) | 0.93 (0.80-1.3) | 0.72 (0.46-0.99) | 0.55 (0.24-0.86) |

*<3 cohorts

HAI-S: Hepatic artery infusion with systemic chemotherapy; TACE-S: Trans-arterial chemoembolization with systemic chemotherapy; TARE-S: Trans-arterial radioembolization with systemic chemotherapy; EHD: extrahepatic disease; FOLFOX (folinic acid, 5-flourouracil, oxaliplatin); FOLFIRI (folinic acid, 5-fluorouracil, irinotecan); FOLFOXIRI (folinic acid, 5-fluorouracil, oxaliplatin, irinotecan).

Supplemental Table 3 (online only). Pooled estimates for progression-free survival

| **Progression free survival** | **Number of cohorts** | **6-month** | **12-month** | **24-month** | **36-month** |
| --- | --- | --- | --- | --- | --- |
| **All cohorts** |  |  |  |  |  |
| HAI-S | 18 | 0.74 (0.63-0.84) | 0.44 (0.34-0.54) | 0.19 (0.12-0.26) | 0.14 (0.05-0.23) |
| TACE-S | 4 | 0.66 (0.48-0.83) | 0.20 (0.08-0.33) | 0.09 (-0.08-0.27) | 0.03 (-0.04-0.10) |
| TARE-S | 6 | 0.57 (0.41-0.72) | 0.23 (0.07-0.39) | 0.10 (-0.06-0.26) | 0.03 (-0.05-0.11) |
| FOLFOX, FOLFIRI | 12 | 0.69 (0.57-0.81) | 0.30 (0.21-0.39) | 0.12 (0.05-0.18) | 0.07 (0.02-0.12) |
| FOLFOXIRI | 4 | 0.88 (0.77-0.98) | 0.55 (0.37-0.72) | 0.18 (0.07-0.30) | 0.11 (-0.01-0.23) |
| **First-line only** |  |  |  |  |  |
| HAI-S | 6 | 0.91 (0.80-1) | 0.60 (0.43-0.77) | 0.28 (0.13-0.43) | 0.15 (0.04-0.34) |
| TACE-S | 2 | * | * | * | * |
| TARE-S | 2 | * | * | * | * |
| FOLFOX, FOLFIRI | 10 | 0.76 (0.67-0.85) | 0.33 (0.25-0.41) | 0.12 (0.05-0.20) | 0.07 (0.01-0.12) |
| FOLFOXIRI | 4 | 0.88 (0.77-0.98) | 0.55 (0.37-0.72) | 0.18 (0.07-0.30) | 0.11 (-0.01-0.23) |
| **No EHD** |  |  |  |  |  |
| HAI-S | 13 | 0.75 (0.63-0.86) | 0.46 (0.34-0.58) | 0.16 (0.08-0.24) | 0.10 (-0.01-0.20) |
| TACE-S | 1 | * | * | * | * |
| TARE-S | 4 | 0.49 (0.33-0.64) | 0.16 (-0.05-0.38) | 0.01 (-0.25-0.27) | * |
| FOLFOX, FOLFIRI | 10 | 0.72 (0.61-0.82) | 0.32 (0.22-0.42) | 0.08 (0.01-0.16) | 0.03 (-0.05-0.11) |
| FOLFOXIRI | 4 | 0.88 (0.77-0.98) | 0.55 (0.37-0.72) | 0.18 (0.07-0.30) | 0.11 (-0.01-0.23) |
| **No EHD, first-line only** |  |  |  |  |  |
| HAI-S | 5 | 0.90 (0.76-1) | 0.64 (0.46-0.82) | 0.31 (0.14-0.47) | 0.16 (-0.06-0.38) |
| TACE-S | 1 | * | * | * | * |
| TARE-S | 1 | * | * | * | * |
| FOLFOX, FOLFIRI | 9 | 0.74 (0.65-0.84) | 0.34 (0.24-0.44) | 0.09 (0.01-0.16) | 0.03 (-0.05-0.11) |
| FOLFOXIRI | 4 | 0.88 (0.77-0.98) | 0.55 (0.37-0.72) | 0.18 (0.07-0.30) | 0.11 (-0.01-0.23) |

*<3 cohorts

HAI-S: Hepatic artery infusion with systemic chemotherapy; TACE-S: Trans-arterial chemoembolization with systemic chemotherapy; TARE-S: Trans-arterial radioembolization with systemic chemotherapy; EHD: extrahepatic disease; FOLFOX (folinic acid, 5-flourouracil, oxaliplatin); FOLFIRI (folinic acid, 5-fluorouracil, irinotecan); FOLFOXIRI (folinic acid, 5-fluorouracil, oxaliplatin, irinotecan).

Supplemental Table 4 (online only). Pooled estimates for conversion to resection and response rate

| **Outcome** | **Number of cohorts** | **Conversion to resection** | **Number of cohorts** | **Objective response rate** |
| --- | --- | --- | --- | --- |
| **All cohorts** |  |  |  |  |
| HAI-S | 20 | 0.31 (0.21-0.41) | 28 | 0.49 (0.41-0.57) |
| TACE-S | 3 | 0.20 (0.04-0.36) | 9 | 0.45 (0.31-0.60) |
| TARE-S | 1 | * | 6 | 0.45 (0.24-0.65) |
| FOLFOX, FOLFIRI | 14 | 0.35 (0.24-0.47) | 13 | 0.50 (0.41-0.59) |
| FOLFOXIRI | 4 | 0.53 (0.44-0.61) | 4 | 0.80 (0.67-0.94) |
| **First-line only** |  |  |  |  |
| HAI-S | 3 | 0.36 (0.10-0.62) | 6 | 0.62 (0.43-0.81) |
| TACE-S | 1 | * | 2 | * |
| TARE-S | 0 | * | 2 | * |
| FOLFOX, FOLFIRI | 12 | 0.39 (0.26-0.51) | 11 | 0.55 (0.47-0.62) |
| FOLFOXIRI | 4 | 0.53 (0.44-0.61) | 4 | 0.80 (0.67-0.94) |
| **No EHD** |  |  |  |  |
| HAI-S | 18 | 0.32 (0.22-0.42) | 18 | 0.56 (0.48-0.64) |
| TACE-S | 1 | * | 5 | 0.42 (0.30-0.53) |
| TARE-S | 1 | * | 2 | * |
| FOLFOX, FOLFIRI | 11 | 0.33 (0.21-0.44) | 10 | 0.51 (0.42-0.60) |
| FOLFOXIRI | 4 | 0.53 (0.44-0.61) | 4 | 0.80 (0.67-0.94) |
| **No EHD, first-line only** |  |  |  |  |
| HAI-S | 3 | 0.36 (0.10-0.62) | 5 | 0.56 (0.36-0.76) |
| TACE-S | 1 | * | 1 | * |
| TARE-S | 1 | * | 1 | * |
| FOLFOX, FOLFIRI | 10 | 0.34 (0.22-0.46) | 9 | 0.53 (0.45-0.62) |
| FOLFOXIRI | 4 | 0.53 (0.44-0.61) | 4 | 0.80 (0.67-0.94) |

*<3 cohorts

HAI-S: Hepatic artery infusion with systemic chemotherapy; TACE-S: Trans-arterial chemoembolization with systemic chemotherapy; TARE-S: Trans-arterial radioembolization with systemic chemotherapy; EHD: extrahepatic disease; FOLFOX (folinic acid, 5-flourouracil, oxaliplatin); FOLFIRI (folinic acid, 5-fluorouracil, irinotecan); FOLFOXIRI (folinic acid, 5-fluorouracil, oxaliplatin, irinotecan).

Supplemental Table 5 (online only). Risk of bias analysis of Hepatic artery infusion studies using the ROBINS-I tool.

| Items | Bias due to confounding | Bias due to selection of participants | Bias in classification of interventions | Bias due to deviation from intended interventions | Bias due to missing data | Bias in measurement of outcomes | Bias in selection of reported result | **Overall** |
| --- | --- | --- | --- | --- | --- | --- | --- | --- |
| *Zelek 2003* | Low | Mod | Low | Low | Low | Low | Low | Mod |
| *Ducreux 2005* | Low | Mod | Low | Low | Low | Low | Low | Mod |
| *Mukai 2006* | Low | Ser | Low | Low | Low | Low | Mod | Ser |
| *Kim 2006* | Low | Mod | Low | Low | Low | Low | Mod | Mod |
| *Gallagher 2006* | Low | Mod | Low | Low | Low | Low | Low | Mod |
| *Carnaghi 2007* | Low | Mod | Low | Low | Low | Low | Low | Mod |
| *Boige 2008* | Low | Mod | Low | Low | Low | Low | Low | Mod |
| *Idelevich 2009* | Low | Mod | Low | Low | Low | Low | Low | Mod |
| *Kemeny 2009* | Low | Mod | Low | Low | Low | Low | Low | Mod |
| *Seki 2009* | Low | Ser | Low | Low | Low | Low | Low | Ser |
| *Goere 2010* | Low | Low | Low | Low | Low | Low | Low | Low |
| *Lee 2011* | Low | Ser | Low | Low | Mod | Low | Mod | Ser |
| *Samaras 2011* | Low | Mod | Low | Low | Low | Low | Low | Mod |
| *Yamaguchi 2011* | Low | Ser | Low | Low | Low | Low | Low | Ser |
| *Arai 2012* | Low | Mod | Low | Low | Low | Low | Mod | Low |
| *Ammori 2012* | Low | Low | Low | Low | Low | Low | Low | Low |
| *Chen 2012* | Low | Mod | Low | Low | Low | Low | Low | Mod |
| *D’Angelica 2015* | Low | Mod | Low | Low | Low | Low | Low | Mod |
| *Qiang 2015* | Low | Ser | Low | Low | Low | Low | Low | Ser |
| *Volovat* | Low | Mod | Low | Low | Low | Low | Mod | Mod |
| *Levi 2016* | Low | Low | Low | Low | Low | Low | Low | Low |
| *Dhir 2016* | Low | Mod | Low | Low | Low | Low | Low | Mod |
| *Cercek 2016* | Low | Low | Low | Low | Low | Low | Mod | Low |
| *Guo 2017* | Low | Mod | Low | Low | Low | Low | Mod | Mod |
| *Dhir 2017* | Low | Low | Low | Low | Low | Low | Mod | Low |
| *Pak 2018* | Low | Low | Low | Low | Low | Low | Low | Low |
| *Boileve 2020* | Low | Low | Low | Low | Low | Low | Low | Low |
| *Muaddi 2021* | Low | Low | Low | Low | Low | Low | Low | Low |
| *Cao 2022* | Low | Low | Low | Low | Low | Low | Low | Low |
| *O’Leary 2022* | Low | Mod | Low | Low | Low | Low | Mod | Mod |
| *Walker 2022* | Low | Ser | Low | Low | Low | Low | Low | Ser |

Low: low risk of bias; Mod: moderate risk of bias; Ser: serious risk of bias

Supplemental Table 6 (online only). Risk of bias analysis of transhepatic chemoembolization studies using ROBINS-I tool

| Items | Bias due to confounding | Bias due to selection of participants | Bias in classification of interventions | Bias due to deviation from intended interventions | Bias due to missing data | Bias in measurement of outcomes | Bias in selection of reported result | Overall |
| --- | --- | --- | --- | --- | --- | --- | --- | --- |
| *You 2006* | Low | Mod | Low | Low | Low | Low | Low | Mod |
| *Akinwande 2013* | Low | Low | Low | Low | Low | Low | Mod | Low |
| *Iezzi 2015* | Low | Ser | Low | Low | Low | Low | Mod | Ser |
| *Yu 2016* | Low | Low | Low | Low | Low | Low | Mod | Low |
| *Pernot 2020* | Low | Low | Low | Low | Low | Low | Low | Low |
| *Cao 2021* | Low | Low | Low | Low | Low | Low | Mod | Low |
| *Liu 2021* | Low | Low | Low | Low | Low | Low | Low | Low |

Low: low risk of bias; Mod: moderate risk of bias; Ser: serious risk of bias

Supplemental Table 7 (online only). Risk of bias analysis of transhepatic radioembolization studies using ROBINS-I tool

| Items | Bias due to confounding | Bias due to selection of participants | Bias in classification of interventions | Bias due to deviation from intended interventions | Bias due to missing data | Bias in measurement of outcomes | Bias in selection of reported result | Overall |
| --- | --- | --- | --- | --- | --- | --- | --- | --- |
| *Hazel 2004* | Low | Ser | Low | Low | Low | Low | Mod | Ser |
| *Hendlisz 2010* | Low | Ser | Low | Low | Low | Low | Low | Ser |
| *Nac 2010* | Low | Low | Low | Low | Low | Low | Mod | Low |
| *Kosmider 2011* | Low | Ser | Low | Low | Low | Low | Mod | Ser |
| *Chua 2011* | Low | Low | Low | Low | Low | Low | Mod | Low |
| *Schonewolf 2014* | Low | Mod | Low | Low | Low | Low | Mod | Mod |
| *Wasan 2017* | Low | Low | Low | Low | Low | Low | Mod | Low |
| *Dhir 2018* | Low | Low | Low | Low | Low | Low | Mod | Low |
| *Mulcahy 2021* | Low | Low | Low | Low | Low | Low | Mod | Low |

Low: low risk of bias; Mod: moderate risk of bias; Ser: serious risk of bias

Supplemental Table 8 (online only). Risk of bias analysis of multiagent chemotherapy studies using ROBINS-I tool

| Items | Bias due to confounding | Bias due to selection of participants | Bias in classification of interventions | Bias due to deviation from intended interventions | Bias due to missing data | Bias in measurement of outcomes | Bias in selection of reported result | Overall |
| --- | --- | --- | --- | --- | --- | --- | --- | --- |
| *Bertolini 2011* | Low | Mod | Low | Low | Low | Low | Low | Mod |
| *Fiorentini 2012* | Low | Low | Low | Low | Low | Low | Low | Low |
| *Ye 2013* | Low | Low | Low | Low | Low | Low | Low | Low |
| *Ychou 2013* | Low | Low | Low | Low | Low | Low | Low | Low |
| *Folprecht 2014* | Low | Low | Low | Low | Low | Low | Low | Low |
| *Gruenberger 2014* | Low | Low | Low | Low | Low | Low | Mod | Low |
| *Dhir 2017* | Low | Low | Low | Low | Mod | Low | Low | Low |
| *Wasan 2017* | Low | Low | Low | Low | Low | Low | Mod | Low |
| *Mise 2020* | Low | Low | Low | Low | Low | Low | Mod | Low |
| *Tang 2020* | Low | Low | Low | Low | Low | Low | Low | Low |
| *Okuno 2020* | Low | Mod | Low | Low | Low | Low | Low | Mod |
| *Dzunic 2020* | Low | Low | Low | Low | Low | Low | Low | Low |
| *Liu 2021* | Low | Low | Low | Low | Low | Low | Low | Low |
| *Hu 2021* | Low | Low | Low | Low | Low | Low | Low | Low |

Low: low risk of bias; Mod: moderate risk of bias; Ser: serious risk of bias

Supplemental Table 9 (online only). GRADE approach to ascertain certainty of evidence.

| **GRADE Certainty assessment** | | | | | | |
| --- | --- | --- | --- | --- | --- | --- |
| **Participants  (studies)** | **Risk of bias** | **Inconsistency** | **Indirectness** | **Imprecision** | **Publication bias** | **Overall certainty of evidence** |
|  |  |  |  |  |  |  |
| **Weighted overall and progression free survival probabilities at 6-, 12-, 24-, and 36-months** | | | | | | |
| HAI-S (1691 patients; 32 studies)  12 clinical trials (37.5%) | not serious | not serious | not serious | not serious | N/A | ⨁⨁◯◯ LOW |
| TACE-S (331 patients; 7 studies)  3 clinical trials (42.8%) | not serious | not serious | not serious | not serious | N/A | ⨁⨁◯◯ LOW |
| TARE-S (1036 patients; 9 studies)  4 clinical trials (44.4%) | not serious | not serious | not serious | not serious | N/A | ⨁⨁◯◯ LOW |
| CT (1712 patients; 14 studies)  12 clinical trials (81.8%) | not serious | not serious | not serious | not serious | N/A | ⨁⨁⨁◯ MODERATE |

HAI-S: Hepatic artery infusion with systemic chemotherapy; TACE-S: Trans-arterial chemoembolization with systemic chemotherapy; TARE-S: Trans-arterial radioembolization with systemic chemotherapy; CT: Multiagent chemotherapy
